# Positive but insufficient: ghana's attempt at eradicating period-related school absenteeism through sanitary pads provision for schoolgirls

**DOI:** 10.3389/frph.2026.1869076

**Published:** 2026-07-08

**Authors:** Godfred Bonnah Nkansah, Carlos Vega, Esther Amofa-Adade

**Affiliations:** 1 Kofi Annan International Peacekeeping Training Centre (KAIPTC), Accra, Ghana; 2 Szechenyi Istvan Egyetem, Győr, Hungary

**Keywords:** bullying, menstrual discomfort, menstruation, period-related absenteeism, sanitary pads, WASH

## Abstract

**Objectives:**

This study reassesses the dominant “pad deficit” explanation for period-related school absenteeism in Ghana by identifying the key factors driving girls' absence during menstruation and assessing perceptions of parents and teachers on the national free sanitary pad policy.

**Methods:**

A mixed-methods approach was employed, combining survey data from over 1,300 adolescent school girls with focus group discussions involving teachers and parents. Descriptive statistics and a supplementary ordered logistic regression model were used to analyze the relative importance and independent effects of reported constraints, while thematic analysis was used to assess the views of stakeholders on period-related school absenteeism.

**Results:**

Among school girls, menstrual discomfort emerged as the most frequently cited reason for absenteeism, followed by inadequate sanitation conditions and limited access to sanitary products, while teachers and parents most frequently attributed absenteeism to sanitary pad deficit. Supplementary regression results confirm that menstrual discomfort outweighs the effects of pad access, WASH infrastructure, and social factors. Stakeholder perceptions suggest that the free pad initiative addresses only part of the problem.

**Conclusion:**

Findings challenge the dominant sanitary pad deficit explanations of period-related absenteeism among school girls in low-income settings, and highlight the need for an integrated, ecosystem-based response that prioritizes biological, material, infrastructural, and socio-cultural constraints to addressing period-related absenteeism among school girls. The study accordingly reconceptualizes period-related absenteeism by positioning menstrual discomfort, rather than material access, as the central predictor of school absence.

## 1 Introduction

Girls' absence from school during menstruation or their periods has often been approached as an individual concern, addressed within households and left largely outside formal educational discourse. Recent studies across Sub-Saharan Africa (SSA) suggest, however, that this framing obscures the broader conditions that influence whether girls can remain in school during their periods. Research from Ethiopia, Uganda, Kenya, Malawi, Nigeria, The Gambia, and Ghana shows that period-related absenteeism is closely tied to a multiplicity of factors, including ways in which the individual, schools, and communities respond to menstruation, not just the menstruation itself, or the inability of a female student to secure a sanitary pad (1–4). Therefore, while a well-meaning government's intervention to address period-related school absenteeism would conventionally center on the provision of sanitary pads as the most critical intervention, this may prove insufficient if an ecosystem approach is not adopted in addressing the challenge.

Across Sub-Saharan Africa, menstruation is noted to disrupt schooling not because it is unexpected, but because educational environments offer limited support for managing it. Studies show that girls often anticipate difficulty during their menstrual periods, adjusting their attendance based on prior experience and perceived risk. In addition to the limited access to affordable and appropriate menstrual materials (5), limited privacy and unreliable water, sanitation and hygiene (WASH) facilities (6, 24, 25), unmanaged menstrual pain (7–9), and the possibility of social exposure and ridicule all contribute to decisions made before a school day begins (3, 10–12). As a result, girls' absence from school during their periods frequently reflects precaution rather than crisis. However, if left unaddressed, menstruation becomes a recurring point of vulnerability within the school year, with absences accumulating gradually instead of appearing as isolated incidents (13).

A major state response to reducing period-related school absenteeism in the Ghanaian context has been the policy of free sanitary pad provision to public basic and senior high schools across the country. First captured as a campaign promise, the newly elected government of President John Dramani Mahama launched the program on the 24th of April 2025, as an augmenting initiative to improve educational outcomes among girls (14). In many parts of the country, girls are documented to miss between one (1) and seven (7) days of school during their period (5, 15). The implementation of the initiative has consequently seen the universal distribution of sanitary pads to public basic and senior high schools for use by all girls, regardless of social and economic class. This, in theory, eliminates the sanitary pad access challenge, largely seen as the main driver of period-related absenteeism, especially among girls from poor socioeconomic backgrounds in the Ghanaian context (15, 16).

This study contributes to the expanding scholarship on the nexus between girls' education and menstrual health in four important ways. First, it challenges the dominant “pad deficit” explanation of period-related absenteeism by showing that sanitary pad provision is not the primary binding constraint for most girls. Second, it provides one of the first large-scale, mixed-method, multi-stakeholder assessments of menstrual-related absenteeism that triangulates the perspectives of students, teachers, and parents within the same institutional context. Third, it assesses the perceptions of key stakeholders―teachers and parents―on the ongoing national policy intervention, allowing us to examine not only why girls miss school, but why a widely adopted policy solution could potentially produce only partial effects. Fourth, it proposes a new conceptual framework which centers firstly on menstrual discomfort, and secondly on sanitary infrastructure, rather than access to sanitary materials, as the main factors associated with period-related absenteeism. Together, the above contributions have important theoretical and policy implications, as they reposition the analytical focus of the ongoing debate away from menstrual material scarcity, toward the broader institutional and personal conditions that shape girls' educational participation.

Although prior studies have examined menstrual health and its impacts among school-going adolescents in the Ghanaian context (5, 15, 17–19), we are not aware of any research that approaches the problem from a holistic ecosystem perspective that systematically triangulates the views of students, teachers, and parents to determine the most critical drivers of absenteeism. Additionally, at the time of submitting this manuscript, we are not aware of any academic study that has empirically assessed the perceptions of parents and teachers on the government's free sanitary pad intervention. Following this introduction, the paper continues with the methodological approach adopted for the study. This is followed by the results section. The paper concludes with discussions on the theoretical and policy implications of the findings.

## 2 Materials and methods

### 2.1 Research design and approach

The study adopted a mixed-methods design, combining a quantitative survey with students and qualitative focus group discussions (FGDs) with parents and teachers. We considered the mixed-method design as more robust than relying solely on either quantitative or qualitative strategies, as it provides both breadth and depth in data collection and analysis. While the survey enabled the collection of data from a large sample of schoolgirls, the FGDs provided insights into the underlying mechanisms that explain how and why period poverty contributes to absenteeism among girls across different regions of Ghana. The integration of these methods thus offered the advantages of triangulation, thereby strengthening the internal validity and reliability of the findings (20, 21).

### 2.2 Sampling design and procedure: survey

We adopted a multistage sampling strategy for the study. First, we adopted the ecological stratification of Ghana into the three zones to capture possible socioeconomic and cultural nuances that could help explain disparities or patterns in the data. Ghana is grouped into three broad ecological zones: the Northern Savanna, the Middle Forest, and the Coastal Belt. These ecological zones do not strictly align with administrative regions, but they provide a useful framework for stratification given their distinct socioeconomic and cultural characteristics. Ghana currently has 16 administrative regions, which cut across the three ecological zones. Within each zone, one administrative region was selected using convenience sampling on the basis that similar Ghana Education Service (GES) administrative structures and school systems operate across the country. Accordingly, the Northern, Ashanti, and Central Regions were selected to represent the three zones.

Within each selected region, two administrative districts were conveniently sampled in consultation with the GES^1^. From each district, three to four basic schools were then conveniently sampled for participation. The basic school system in Ghana comprises primary (6 years), junior high (3 years), and senior high school (3 years), serving students aged approximately 6 to 18 years. Although primary and junior high enrollment rates are relatively high, the introduction of the government's Free Senior High School Policy has significantly expanded access at the secondary level (22). Senior high schools are, therefore, generally more populated than primary or junior high schools. To avoid overrepresentation of secondary school students, the study ensured that in each selected district, one senior high school and at least two primary or junior high schools were included. In each selected school, girls from Primary Six (average age 11 years) through to the final year of Senior High School (average age 18 years) were conveniently sampled for participation. In smaller schools, this often meant including all eligible girls present, while in larger schools, particularly senior high schools, participants were drawn from available classes at the time of the visit. The choice of convenience sampling was informed by constraints on time, finance, and physical accessibility to some of the schools.

### 2.3 Sampling approach: focus group discussions

In total, nine FGDs were conducted across the three selected regions, with three discussions per region. Of these, six FGDs were held with teachers and three with parents. To account for gender dimensions, two FGDs consisted exclusively of female participants, while the remaining seven were mixed-gender groups. Among the teacher FGDs, three involved teachers at the primary/junior high school (JHS) level, while the other three included senior high school (SHS) teachers, ensuring balanced representation across different levels of the education system. Teacher participants were recruited with the assistance of school head teachers, who invited available and willing teachers to take part. Group sizes for teacher FGDs ranged from four to nine participants. For parent FGDs, invitations were extended to all parents of students in the selected schools through the school administrations. Participation was voluntary, and those who responded were included in the discussions. Group sizes ranged from seven to twenty parents. Although the group with twenty participants was unusually large for an FGD, moderators employed participatory and inclusive facilitation techniques to ensure active engagement of all participants throughout the discussion.

### 2.4 Data collection

A total of 1,500 questionnaires were printed and administered across the three regions. In Ghana, students in public schools are not permitted to use mobile phones in school, while access to computers and reliable internet connectivity remains limited in many schools. Consequently, in consultation with the GES, printed questionnaires were deemed the most feasible and pragmatic option for the study.^2^ The questionnaires were self-administered in English, under the close supervision of the research team, with teachers providing additional support where necessary. Each survey session lasted an average of 30 min. Among other questions, students were asked whether they had *access to sanitary pads, were comfortable with their menstrual products, had sanitary facilities in their schools, the state of the sanitary facilities, have ever been bullied or stigmatized due to their period, experience discomfort/pain during their period, whether they miss school during their periods, and importantly, the main reason why they miss school during their period.*

For the six teacher FGDs, moderation was carried out by members of the research team. The three parent FGDs were co-facilitated by a research team member and a local translator, as discussions were conducted in the predominant local dialects of the respective communities. Each FGD lasted for an average of 90 min. To ensure accurate documentation, a member of the research team not involved in moderating systematically took notes on a laptop during the sessions. The FGDs asked questions about *the challenges girls face during their menstrual periods, the impacts of such challenges on their schooling, the role of schools, the government, parents/communities in addressing period related absenteeism,* and *strategies parents and teachers recommend for improved support for girls to help reduce absenteeism*.

### 2.5 Data analysis

Responses from the questionnaires were primarily analysed using descriptive statistics such as bar charts, cross-tabulations and frequency tables using STATA 17 software^3^. Given the focus of the study, however, the analysis excluded data on girls who reported that they had not yet experienced menarche. This brought the final sample size used for the analysis to 1,346 girls (*N* = 1,346). For the FGDs, notes were analysed using a thematic approach, highlighting the thematic findings, particularly those related to the causal mechanisms that help explain period-related school absenteeism. Although the primary analysis in this paper is descriptive due to the paper's interest in assessing stakeholder perspectives and ranking of constraints, we estimated a supplementary logistic regression model to assess whether the observed patterns in the descriptive data and FGDs hold when covariates are considered simultaneously. The ordinal logit equation for missing school during menstrual cycle is specified in Equations 1 and 2 below:

$$\begin{array}{rl} Y_{i}^{*}= & \beta_{0\;}+\beta_{1\;}Age_{i}\;+\;\beta_{2\;}ASP_{i}\;+\;\beta_{3\;}CST_{i}+\;\beta_{4\;}WDB_{i}+\;\\ & \beta_{5\;}ACRS_{i}\;+\;\beta_{6\;}AHFS_{i}\;+\;\beta_{7\;}ExB_{i}\;+\;\beta_{8\;}SSup_{i}+\;\\ & \beta_{9\;}SSDem_{i}\;+\;\beta_{10\;}FASS_{i}\;+\;\beta_{11\;}MDisc_{i}+\;\varepsilon_{i\;} \end{array}$$ (1)

The observed categories are:

$$Y_{i\;\;\;}=\;\{\begin{array}{c} 1\;\;\;\;\;\;\;\;\;\;\;\;\;\;\;\;\;\;\;\;\;\;\;\;\;\;\;\;\;\;\;No\;\;\;\;\\ 2\;\;\;\;\;\;\;\;Yes,\;occasionally\;\\ 3\;\;\;\;\;\;\;\;\;\;\;\;Yes,\;every\;cycle \end{array}$$

The odds ratio for missing school during menstrual cycle is specified as:

$$\begin{array}{rl} \frac{P(Y_{i\;\;\;}\leq j)}{P(Y_{i}\;>j)}= & \exp[\mu_{j}-(\beta_{1\;}Age_{i}+\beta_{2\;}ASP_{i}+\beta_{3\;}CST_{i}+\beta_{4\;}WDB_{i}\\ & +\;\beta_{5\;}ACRS_{i}+\;\beta_{6\;}AHFS_{i}+\beta_{7\;}ExB_{i}+\;\beta_{8\;}SSup_{i}\\ & +\;\beta_{9\;}SSDem_{i}+\beta_{10\;}FASS_{i}+\;\beta_{11\;}MDisc_{i})] \end{array}$$ (2)

j=1,2

Where;

$Y_{i\;\;\;}$ denotes the likelihood of missing school during every menstrual cycle by the individual girl,

$\mathrm{Ag}e_{i}$ is the age of the individual girl,

$\mathrm{AS}P_{i}\;$ represents individual girl's access to sanitary pad,

$\mathrm{CS}T_{i}$ represents cleanliness of school toilet of the individual girl,

$\mathrm{WD}B_{i}$ represents availability of waste disposal bin in the school of the individual girl,

$\mathrm{ACR}S_{i}$ represents availability of changing room in the school of the individual girl,

$\mathrm{AHF}S_{i}\;$ reperents availability of handwashing facilities in the school of the individual girl,

$\mathrm{Ex}B_{i}\;$ represents individual girl's experience of bullying due to menstruation,

$\mathrm{SSu}p_{i}$ represents school support of the individual girl,

$SSDem_{i}$ represents individual girl's staining in school during menstruation,

$\mathrm{FAS}S_{i}$ represents individual girl's feeling about staining in school,

$\mathrm{MDis}c_{i}$ represents individual girl's menstrual discomfort,

$\mu_{j}$ denotes the threshold parameters.

$\beta_{1},\;\beta_{2\;}\ldots \beta_{11\;}$ are the coefficients.

### 2.6 Ethical considerations

Given that the study involved school-aged children, particular care was taken to ensure adherence to ethical standards throughout the data collection process. Cognisant of the fact that students fall under the authority of the GES during school hours, formal permission to access schools was sought from the GES. The Monitoring, Evaluation, Research and Learning Division and the Schools and Instructions Division, of the GES vetted and approved the study, including all data collection tools, selected districts and schools for the study. Additionally, a GES focal person at the level of a National Divisional Director liaised directly with Regional and District Directors of education, as well as school administrators, to facilitate smooth and transparent operations. Furthermore, all student participants were provided with an initial briefing on the objectives of the study prior to their involvement. The survey questionnaires also contained an introductory statement explaining the purpose of the research and included a section requesting informed consent. Students indicated their voluntary agreement to participate by ticking “yes” or “no” before completing the questionnaire. Furthermore, strict measures were taken to protect confidentiality and privacy. The questionnaires did not include personally identifiable information about individual students or schools. The only geographic identifier recorded was the administrative district, which ensured that individual participants and institutions remained anonymous.

For the FGDs, verbal informed consent was obtained from all participants, teachers and parents, before the sessions commenced. Participation was entirely voluntary, and participants were assured of confidentiality and the non-attributable use of their responses. Through these measures, the study ensured compliance with ethical research standards while protecting the rights, privacy, and well-being of all participants.

## 3 Results

We present the results of the study below, beginning with the outcomes of the FGDs with parents and teachers, followed by the quantitative analysis of students' responses to the questionnaires.

### 3.1 Thematic analysis of FGDs

The following are the major issues drawn from thematic analysis of FGD responses from parents and teachers. They are briefly discussed under the following major headings:

#### 3.1.1 Main challenges schoolgirls face during menstruation

Teachers and parents consistently reported that many girls are unable to afford sanitary pads. As a result, they resort to using cloth, toilet paper, or other improvised and unhygienic materials. This not only causes physical discomfort but also exposes them to stigma and embarrassment among peers. Additionally, severe cramps, headaches, vomiting, and loss of concentration were reported as common challenges that disrupt girls’ learning during menstruation. None of the schools visited had infirmaries or first aid services that could provide relief, leaving girls to cope on their own. Consequently, even those who attend school during their periods often end up sleeping in class or excusing themselves for long periods of the day, reducing their ability to fully participate in lessons. A teacher remarked as follows:

“You will sometimes find them with their heads on the table while teaching; they cannot concentrate because of the cramps”

Also, many schools lacked functional washrooms or changing rooms where girls could comfortably and privately change their pads. In several cases, sanitary facilities were either absent, inadequate, shared with the wider community, or in a state of disrepair. Faced with these conditions, many girls preferred to stay at home during some days of their menstrual cycle rather than risk embarrassment at school. Relatedly, the teachers’ FGDs also reported that a number of girls, especially those who begin menstruation at an early age, report lacking adequate knowledge and preparedness for menstrual hygiene management. Parents rarely provided them with timely information about menstruation before menarche, leaving them unprepared and anxious when they first experienced their periods.

#### 3.1.2 Impact of menstrual health challenges on school attendance and participation

Teachers reported that many girls miss between two and five days of school each month as a result of menstrual challenges. Severe cramps, lack of access to sanitary pads, absence of private changing facilities on campus, and feelings of embarrassment often force them to stay at home during their periods. Additionally, even when girls attend school while menstruating, their participation in class activities tends to be limited. Pain, fear of staining their clothes, or the possibility of being mocked discourage them from speaking or engaging fully. Some teachers misinterpret this reduced participation as inattentiveness or lack of seriousness, and in some cases punish the girls, further compounding their discomfort and marginalisation. An FGD participant remarked as follows:

“Sometimes, especially some of the male teachers who do not know what is happening to the girls, resort to punishment when they find them sleeping in class: they don’t know that it is because of menstrual pains and discomfort”

Furthermore, menstruation often subjects girls to stress, shyness, and teasing by male peers, all of which undermine their confidence and willingness to learn. This was noted by the FGD to be especially challenging for younger girls and those who have only recently begun menstruating, as they are more vulnerable to embarrassment and negative peer pressure.

#### 3.1.3 Common misconceptions and cultural beliefs

Both parents and teachers in the FGDs noted that cultural and religious beliefs surrounding menstruation continue to restrict girls’ participation in everyday activities. In some communities, menstruating girls and women are discouraged or prevented outright from cooking, fetching water, or engaging in religious and community practices, reinforcing a sense of isolation during their periods. Also importantly, menstruation is still widely associated with impurity, bad odor, and taboo. Such perceptions perpetuate stigma, causing girls to feel ashamed and excluded, particularly in school and community settings where openness about menstruation is already limited. Additionally, across several communities, the FGD reported that persistent myths continue to shape negative attitudes toward menstruation. Some believe that menstrual cramps automatically stop after childbirth, while others regard menstrual blood as harmful or dangerous. These misconceptions reinforce misinformation, prevent healthy conversations, and contribute to the broader stigma surrounding menstrual health.

#### 3.1.4 Role of schools in supporting girls

The FGD participants reported that some schools, often with the support of NGOs, provide sanitary pads to girls in need. As earlier reported, the government has also introduced a policy to supply free sanitary pads to all girls in public basic schools nationwide. However, implementation has been uneven. While a few of the schools visited during the study had received their quarterly allocations, others were still waiting for supplies, despite the policy having been in effect since the second quarter of 2025. Also importantly, awareness-raising efforts are mostly carried out through health clubs, teachers, and special programs such as Menstrual Hygiene Day. Gender Focal Persons, Girl-Child Coordinators, and Counseling Coordinators typically lead these initiatives. A teacher remarked:

“We sensitize them on menstrual hygiene; every school has a girl-child coordinator and they speak to the girls”.

However, such education is irregular, occurring mainly during commemorative events such as World Menstrual Hygiene Day, and, therefore, fails to provide girls with consistent, ongoing guidance on menstrual hygiene management. Substantively, institutional responses vary widely across schools. Some have established structured health or girls’ clubs to address menstrual hygiene issues, while others rely primarily on the voluntary efforts of individual teachers.

#### 3.1.5 Needed school facility improvements

The lack of private, functional washrooms and changing rooms was identified as one of the most pressing challenges for schoolgirls. Through the FGDs, the study found that in many schools, existing toilets are either dysfunctional, co-shared with the wider community, or entirely unavailable. This lack of facilities forces many girls, particularly those with heavy flows who need to change pads multiple times during the school day, to stay home to avoid embarrassment. A teacher remarked as follows during a FGD session in one of the schools:

“You see the toilet behind the school building [pointing to an old, dilapidated structure]; it is in a very poor shape, yet the children share it with the whole community. You can’t stay there for one minute when you attempt to use it”

According to the teachers, *the absence of proper changing spaces is the second biggest contributor to period-related absenteeism, after availability and cost of sanitary pads*. Parents generally shared in the same ranking, with the cost of sanitary products as the biggest reason their kids stay at home, followed by institutional sanitary infrastructural challenges.

#### 3.1.6 Perceptions of the government's free sanitary pad policy

Teachers in the FGD reported that the distribution of sanitary pads remains the most requested intervention. They, therefore, assessed the government's free sanitary pads intervention as timely and one that addresses the access or availability question. However, the current government supply assumes uniform needs by providing pads suitable for only light to medium flows. This leaves girls with heavy flows dependent on the open market, where affordability remains a challenge. To ensure inclusivity, the FGD reported that the policy should recognize the diversity of menstrual experiences and address the reliability question. Provisions should thus recognize the diverse needs of menstruating girls by supplying different grades of pads: light, medium, and heavy flow, so girls can manage their periods with comfort and dignity. Policies should also promote affordability and access outside school settings, ensuring sustainability beyond direct distribution programmes. An FGD participant remarked:

“The pads the government has brought are good, but they are light and not suitable for those with heavy menstrual flows. If your flow is heavy, you need to change the pad every 2–3 h. We need the ones for heavy flows, too”.

Also importantly, the FGD participants reported that government investment in functional washrooms and changing rooms is urgently needed. To the FGD respondents, while the free pad initiative addresses one aspect of menstrual health, it remains incomplete without parallel gender-sensitive infrastructural support, since the lack of private, sanitary facilities continues to be a major factor keeping girls out of school during their periods. Furthermore, the FGD acknowledged the lack of targeted health interventions to address the issue of menstrual pain and discomfort as part of the free sanitary pad initiative. The respondents hence suggested that health services should be integrated into school settings, with trained nurses or health officers available to provide pain relief and medical support for girls experiencing severe cramps or related challenges. Equally important, the current free pad initiative was rolled out without adequate planning for waste management or sensitization on safe disposal. Given the scale of such a universal pad distribution, respondents reported that the absence of an eco-friendly disposal strategy risks environmental pollution from discarded pads and their packaging.

### 3.2 Survey results

We present the results of the survey below, beginning with the descriptive statistics of the sample.

#### 3.2.1 Descriptive statistics of respondents

Table 1 below presents the key sociodemographic indicators from the sample population of school girls. 63% of the respondents were in senior high school. Only 4% of the respondents were below 13 years, the dominant age at which most girls (27%) experienced menarche. By religion, Christians dominated, consistent with Ghana's demographic composition, with 71% Christians.

**Table 1:** Sociodemographic characteristics of girls in the sample population.

Item	Categories	Frequency	Percentage (%)
School level	Junior High School	489	36.57
	Senior High School	848	63.43
Age	Less than 10 yrs	2	0.15
	10–12 yrs	55	4.13
	13–15 yrs	440	33.01
	16–19 yrs	780	58.51
	Above 20 yrs	56	4.20
Religion	Christians	845	63.58
	Muslims	482	36.27
	Other	2	0.15
Menarche	Before 10 yrs	38	2.82
	10 yrs	69	5.13
	11 yrs	126	9.36
	12 yrs	302	22.44
	13 yrs	360	26.75
	14 yrs	245	18.20
	15 yrs	206	15.30

#### 3.2.2 Period-related absenteeism and contributing factors

Respondents were asked whether they miss school during their menstrual period. 68% (914) of the sample reported never missing school, 14% (185) reported missing school always, while 18% (238) reported missing school occasionally. Taken together, 32% (432) of the students surveyed reported period-related absenteeism during their cycles. Regarding the number of days of period-related absenteeism by the affected students, 31% reported missing one school day, 29% missed two school days, while 40% missed three or more school days during their monthly cycles.

**Figure 1:**
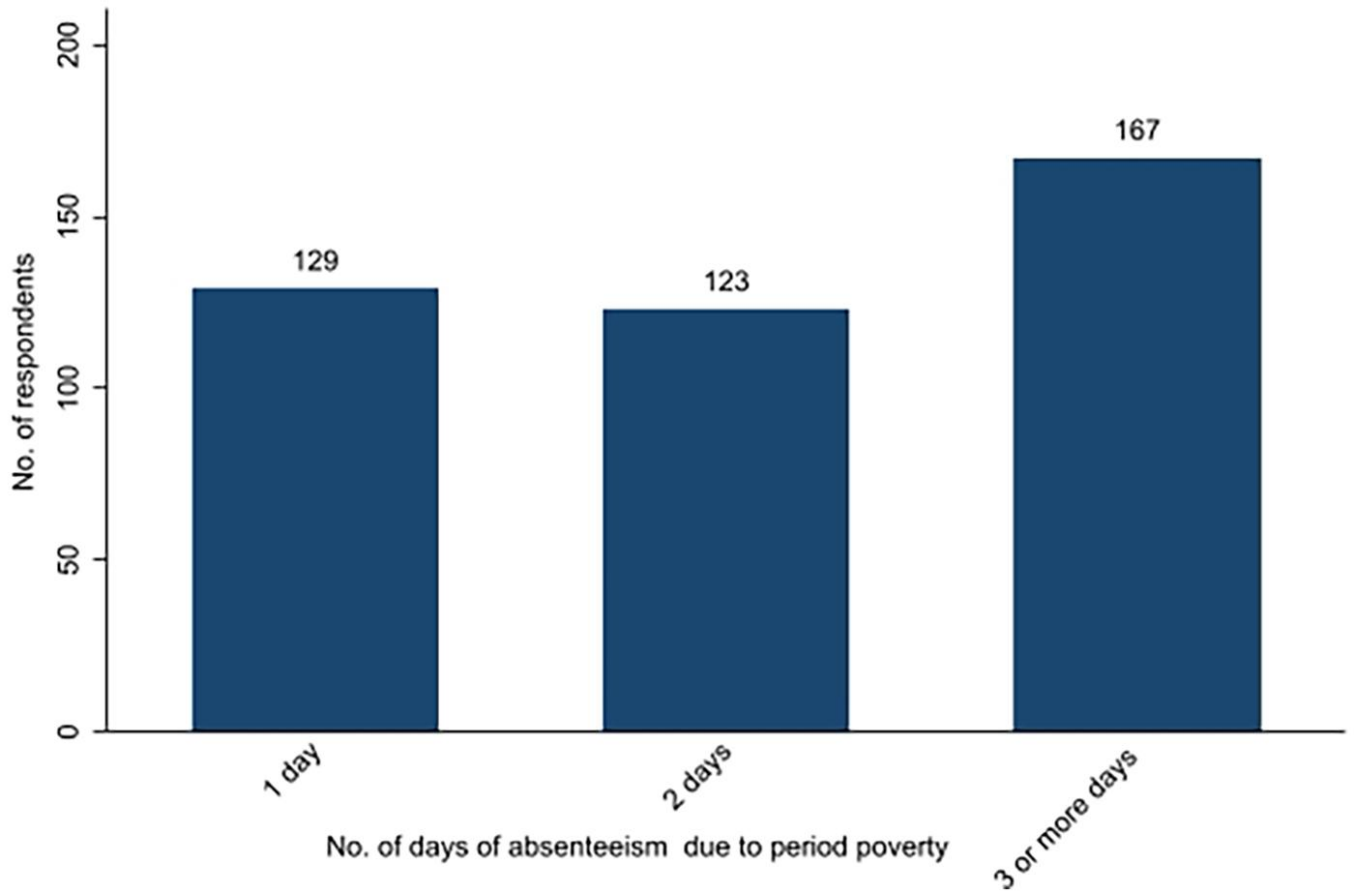
Frequency of period-related absenteeism among girls in sample who report missing school during their cycles every month.

Regarding the main reasons respondents miss school during their monthly periods, the questionnaire provided options in line with the reviewed literature. These included lack of sanitary facilities at school (e.g., disposable bins, washrooms, and flowing water for handwashing), menstrual discomfort (e.g., cramps), fear of staining, fear of bullying, and the lack of sanitary pads. Figure 1 below presents the bar chart of period-related absenteeism among the students.

**Figure 2:**
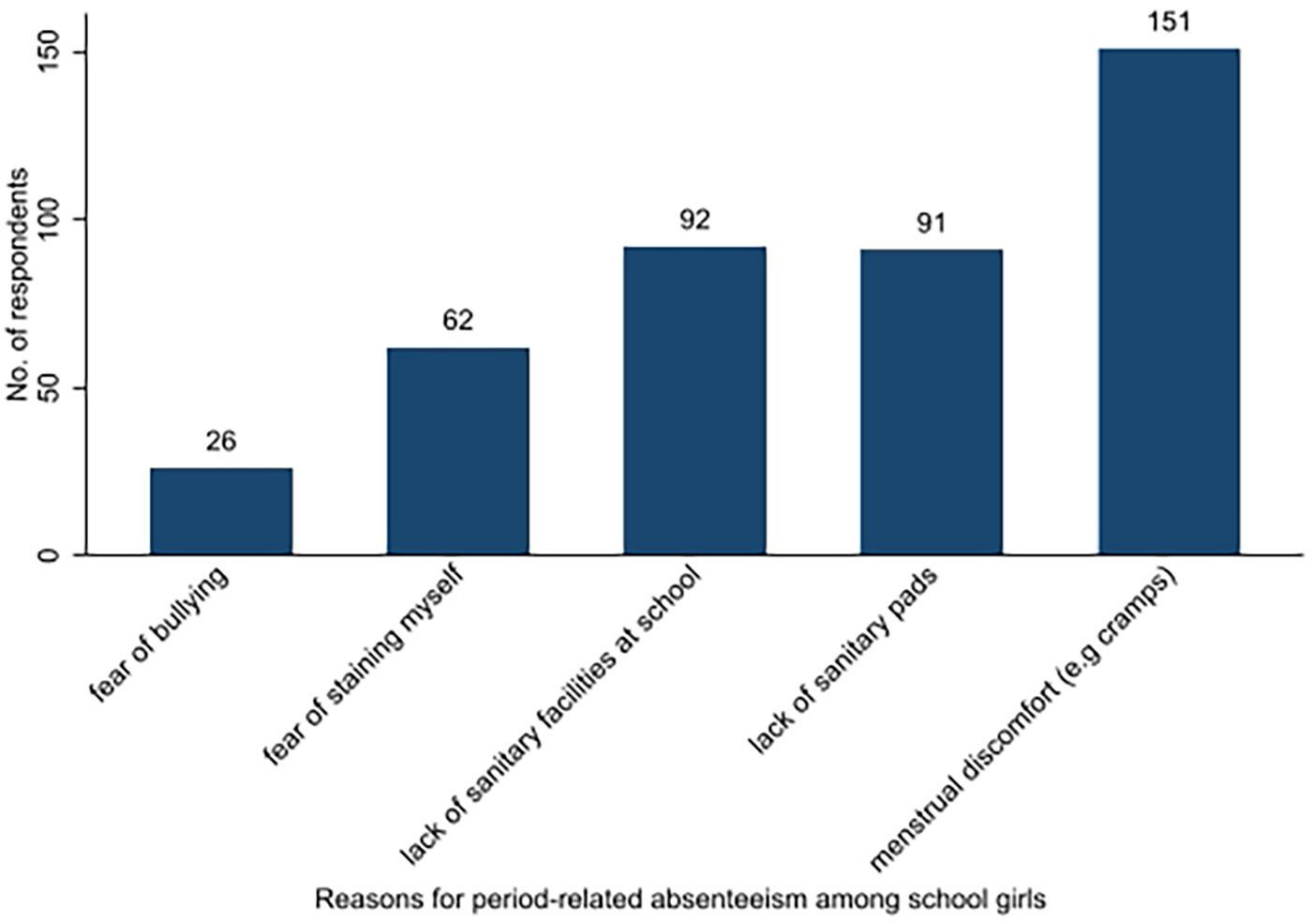
Students’ reasons for missing school during their periods.

Figure 2 below shows that the single most important factor for period-related school absenteeism among girls in the sample is menstrual discomfort, followed by water, sanitation, and hygiene facilities challenges in school, before sanitary pad-related concerns. We probed students’ access to sanitary pads to ascertain the availability of such materials. 52% reported always having access to sanitary pads, 24% have access only sometimes, 7% rarely have access, while 18% had no access to sanitary pads. We then cross-tabulated access to sanitary pads with the main reasons for period-related absenteeism to ascertain what could potentially still keep girls away from school during their cycles, particularly where access to sanitary materials is guaranteed. Table 2 above shows the results of the cross-tabulation. The analysis reveals that even among girls with constant access to sanitary pads, nearly 53% of them may still miss school as a result of menstrual discomfort, followed by 21% who miss school as a result of the sanitation infrastructure in their schools. This suggests the relatively stronger influence of menstrual discomfort in explaining period-related school absenteeism.

**Table 2:** Cross-tabulation of access to sanitary pads and the main reasons for period-related absenteeism.

Access to Sanitary Pads	Fear of bullying	Fear of staining myself	Lack of sanitary facilities at school	Lack of sanitary pads	Menstrual discomfort (e.g cramps)	Total
Always	16	20	37	16	90	179
No	3	13	23	35	16	90
Only sometimes	5	17	24	33	37	116
Rarely	1	12	8	7	7	35
Total	25	62	92	91	150	420

Quite interestingly, the results of the supplementary ordinal logistic regression presented in Table 3 below align with the reported patterns in the descriptive data. Menstrual discomfort emerged as the strongest predictor of period-related absenteeism, among all the other predictors— age, availability, and conditions of sanitation facilities, bullying, the experience and feeling of staining during school hours. Girls who always experience menstrual discomfort have nearly 4 times higher odds of absenting themselves from school during their cycles, compared to those who don't experience menstrual discomfort (OR = 3.65, *p* < 0.001). Even girls who experience menstrual discomfort occasionally show higher odds of moving into more frequent absenteeism (OR = 2.67, *p* < 0.01). Although limited access to sanitary pads was also associated with higher absenteeism (OR = 1.7–1.9), the magnitude of these effects was substantially smaller than those observed for menstrual pain. The other predictors, such as teacher bullying and the availability of disposal facilities, showed moderate associations, while several infrastructural indicators were not statistically significant.

**Table 3:** Results of multivariate ordinal logistic regression for the effect of key predictors of period-related absenteeism (results are presented in *odds ratios* with robust standard errors).

Independent Variables	Dependent Variable Missing school during menstrual cycle (Yes, every cycle)
Age^a^	
10–12	0.192** (0.108)
13–15	0.301** (0.113)
16–19	0.187*** (0.068)
20 and above	0.275** (0.134)
Access to Sanitary Pads^b^	
Only sometimes	1.802** (0.343)
Rarely	1.902* (0.586)
No	1.698** (0.341)
Cleanliness of Toilet^c^	
Somewhat clean	0.460*** (0.101)
Not clean	0.314*** (0.072)
Waste Disposal Bin	
Yes	0.659* (0.113)
Availability of Changing Room in School^d^	
Yes, functional but not comfortable to use	0.949 (0.314)
Yes, but broken and unusable	1.659 (0.587)
No	1.086 (0.246)
Availability of Handwashing Facilities^e^	
Yes, functional but not comfortable to use	1.155 (0.582)
Yes, but broken and unusable	0.864 (0.278)
No	0.643* (0.130)
Experience of Bullying^f^	
Yes, by peers	1.047 (0.219)
Yes, by teachers	1.711* (0.432)
School Support	
Yes	0.812 (0.196)
Staining in School During Menstruation	
Yes	0.857 (0.175)
Feeling about staining in School^g^	
I didn’t feel bad about it	0.799 (0.263)
I felt embarrassed	1.009 (0.272)
Menstrual Discomfort^h^	
Sometimes, but not always	2.671** (0.950)
Yes, always	3.652*** (1.286)
Model Characteristics	
cut1	0.437 (0.259)
cut2	1.518 (0.887)
No. of respondents	853
Log pseudolikelihood	−710.97

Entries are the odds ratios with robust standard errors (in parentheses) for an ordered logistic regression model. Reference category.

a Less than 10 years.

b Yes, I have access to sanitary pads.

c Very clean.

d Yes, we a changing room.

e Yes, we have handwashing facilities.

f No, I don’t experience bullying.

g I didn’t care.

h No, never.

*p* < 0.05.

*p* < 0.01.

*p* < 0.001.

Regarding the frequency of menstrual discomfort, 9% of all girls reported always experiencing it, 39% experience discomfort only sometimes, while 52% do not experience any form of discomfort. On the severity of menstrual discomfort, 24% of girls reported experiencing mild discomfort, 38% reported moderate discomfort, while the remaining 38% also reported severe discomfort. When asked how they manage the discomfort, 23% reported using herbal medicine, 24% use hot bottled water, while 49% use painkillers, while 5% use other methods of pain relief they did not disclose.

**Figure 3:**
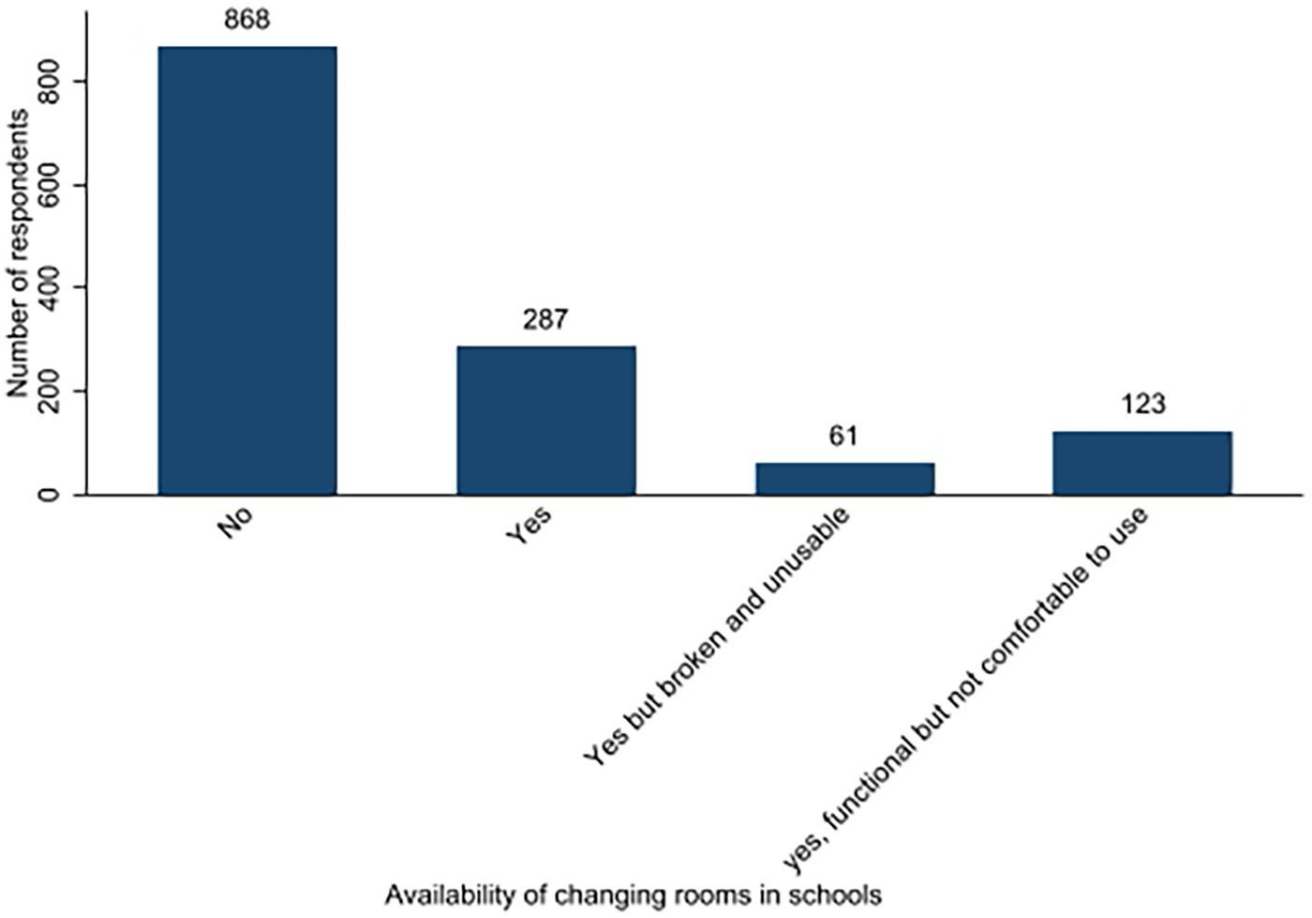
Students’ view on the availability of changing rooms in schools.

**Figure 4:**
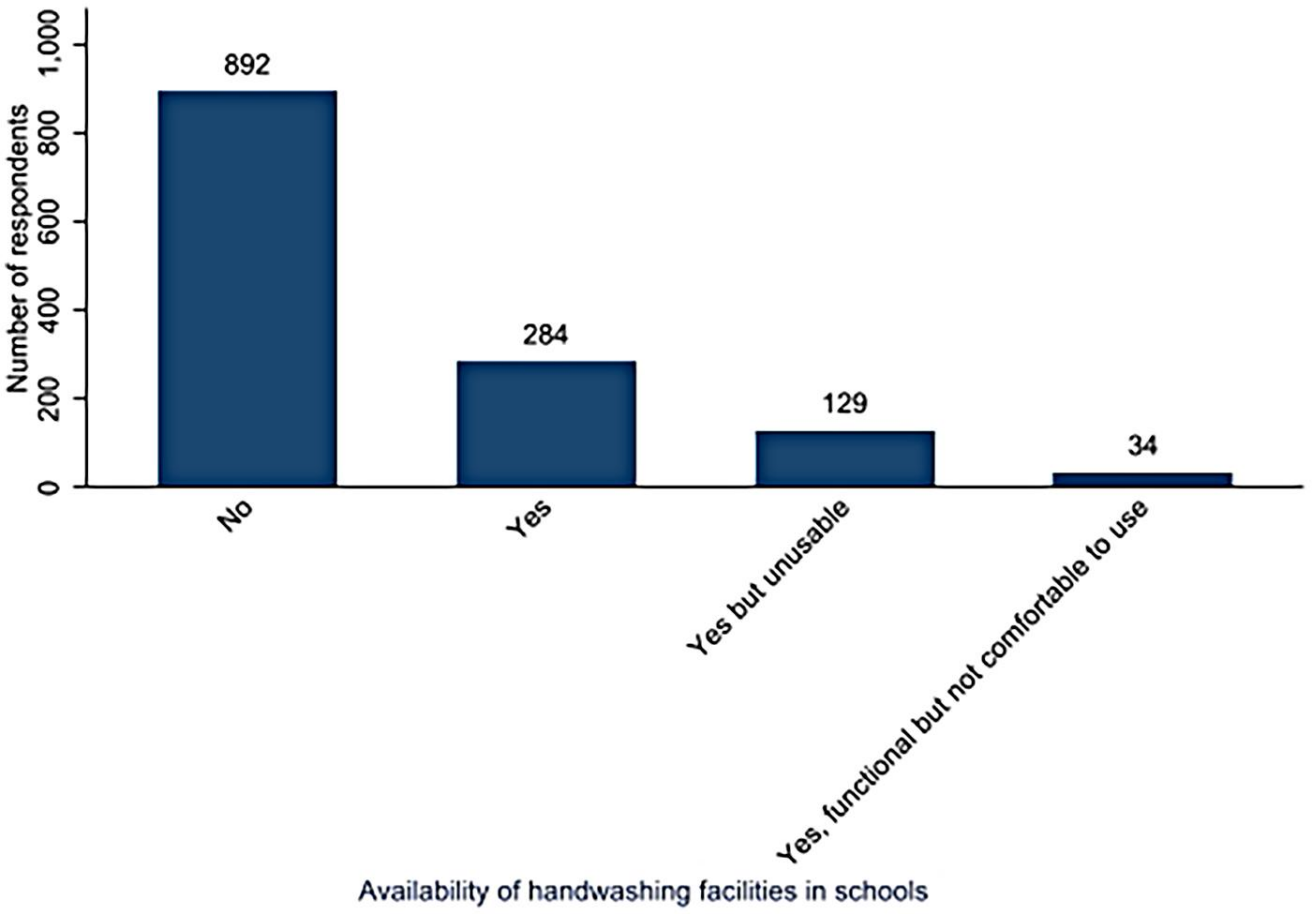
Handwashing facilities in schools.

The questionnaire asked a series of questions related to the availability and functionality of WASH facilities in schools to gain a better understanding of the contributions of sanitation facilities to period-related school absenteeism. On whether their schools have toilet facilities, 46% indicated they have, while 49% described the state of their toilets as uncomfortable to use. 5% did not have toilets in their school. Regarding the cleanliness of the toilets for use, 41% were emphatic in their indication that the toilets were not clean, 40% described their school toilets as somewhat clean, while 19% thought the toilets were very clean, suggesting that 81% of the students surveyed were dissatisfied with the state of the toilet facilities in their schools. Figures 3, 4 below present the results of the analysis of the availability of changing rooms and handwashing facilities in the schools. The results show that changing rooms and handwashing facilities are mostly lacking across schools. Where they are available, a sizeable proportion of the students rate them as unusable and uncomfortable to use, indicating a serious challenge with WASH from the perspective of the students.

## 4 Discussion and conclusion

Our study sought to ascertain, in order of relevance, the core reasons why girls miss school during their menstrual cycles in Ghana, based on the assessments of students, teachers, and parents. Existing studies identify several factors for period-related absenteeism. Of these challenges, access to sanitary materials emerges in some studies as the most important contributor to period-related absenteeism, particularly in the Ghanaian context (15, 16), consequent to which the Government of Ghana has initiated the provision of free sanitary pads to all school-going girls in public educational institutions, from the basic to senior high school level. The second ambition of our study, therefore, aimed to assess the perceptions of parents and teachers on the Government of Ghana's free sanitary pads initiative. We discuss the main findings under the themes below:

### 4.1 Menstrual pain and classroom constraints

Our empirical findings suggest that the core assumption that access to sanitary materials is the main driver of period-related poverty is overstated, at least in the Ghanaian context. Our triangulated results show that pads are not the binding constraints on most girls. Across our sample population, students consistently identified menstrual discomfort and concerns with the state of sanitation infrastructure in their schools as the two principal reasons for absenteeism during their periods. Access to sanitary pads only came third. Importantly, the results also reveal that even among girls who have constant access to sanitary pads, more than half (53%) miss school due to menstrual discomfort (see Table 2). Relatedly, our supplementary ordinal regression analysis of the predictors of period-related absenteeism using the survey data showed that menstrual discomfort was the strongest predictor of absenteeism during a girl's menstrual cycle (see Table 3). The results suggest that product provision alone may not address the participation problem. In other words, pads are a necessary but insufficient condition for school attendance for girls. Our findings, therefore, challenge the dominant materialist framings of period poverty that equate access to sanitary pads with educational inclusion, and suggest a rethinking of the dominant “pad deficit” explanation of period-related absenteeism, in favor of a broader, systems-oriented understanding.

More broadly, the role of menstrual pain and discomfort as an independent determinant of period-related absenteeism has been under-theorised and under-recognised in existing research. Our review of the literature shows that while menstrual discomfort is frequently acknowledged alongside other factors, it is rarely treated as the primary explanatory variable in most research and programme designs [(e.g. (3, 12, 15, 23, 26)]. Yet in this study, menstrual discomfort emerged as the single most frequently cited reason for missing school, even among girls with reliable access to sanitary products. What appears evident, therefore, is that menstrual discomfort operates independently of material constraints, such that in school environments where rest, analgesics, or health services are unavailable, attendance may simply be physically unsustainable for most girls. In this way, interventions that focus narrowly on sanitary products, such as the Government of Ghana's free pad initiative, address only one dimension of the problem, while overlooking or downplaying a key biological reality. Practical steps such as access to pain relief, school-based health services, and flexible classroom arrangements may, therefore, better align policy with girls’ lived bodily experiences and expand the range of effective, actionable solutions available to education planners.

### 4.2 Institutional sanitation barriers

The findings further indicate that school WASH facilities should not be treated as a peripheral or background condition but rather as an institutional gatekeeper of girls’ educational participation, particularly in the Ghanaian context. Across both the survey and focus group data, the absence or dysfunctional state of toilets, changing rooms, and a reliable water supply consistently emerged as critical constraints. Even where sanitary pads are available, girls are unable to manage menstruation with dignity if they lack private, safe, and hygienic spaces to change or clean themselves. Under such conditions, remaining in school during menstruation becomes practically difficult and socially risky. This indicates that the state of sanitation infrastructure determines whether menstrual management is feasible within the school day and largely aligns with past studies, which reported WASH concerns as the most cited reason for period-related absenteeism (e.g., see., 26). We think that conceptually, this finding repositions WASH from being one factor among many to a foundational enabling precondition for attendance. Without adequate and functional facilities, the effectiveness of other interventions, including pad distribution, is inherently limited (See similar arguments in (1, 6)]. We argue, therefore, that the foregoing demonstrates the need to integrate menstrual health considerations into school infrastructure planning and to treat gender-responsive WASH investments as central, rather than complementary, components of education policy.

### 4.3 Adult vs. student perception gap

Equally important, the findings also highlight the divergent views of our stakeholders on the reasons for period-related absenteeism and their potential policy implications. While parents and teachers overwhelmingly identified pad affordability and availability as the principal barriers to attendance, girls themselves emphasised bodily discomfort and the everyday realities of navigating or unusable school sanitation infrastructure. We think that this discrepancy is analytically important. In substantive terms, adult respondents in the study tended to interpret period-related absenteeism through structural or economic lenses, while girls primarily framed their decisions around lived experience, including pain, and the absence of private spaces. This suggests that policies designed primarily around adult policy makers’ perceptions, such as the Ghanaian free sanitary pad initiative, risk misdiagnosing the problem and potentially misallocating resources. Therefore, moving forward, it will be critical to centre girls’ own accounts in both research and intervention design since, as the results of this study show, attendance decisions are not simply responses to resource scarcity, but rather, subjective calculations about whether the school environment feels manageable and safe during menstruation.

### 4.4 Reconceptualizing the theory of change for period-related absenteeism

**Figure 5:**
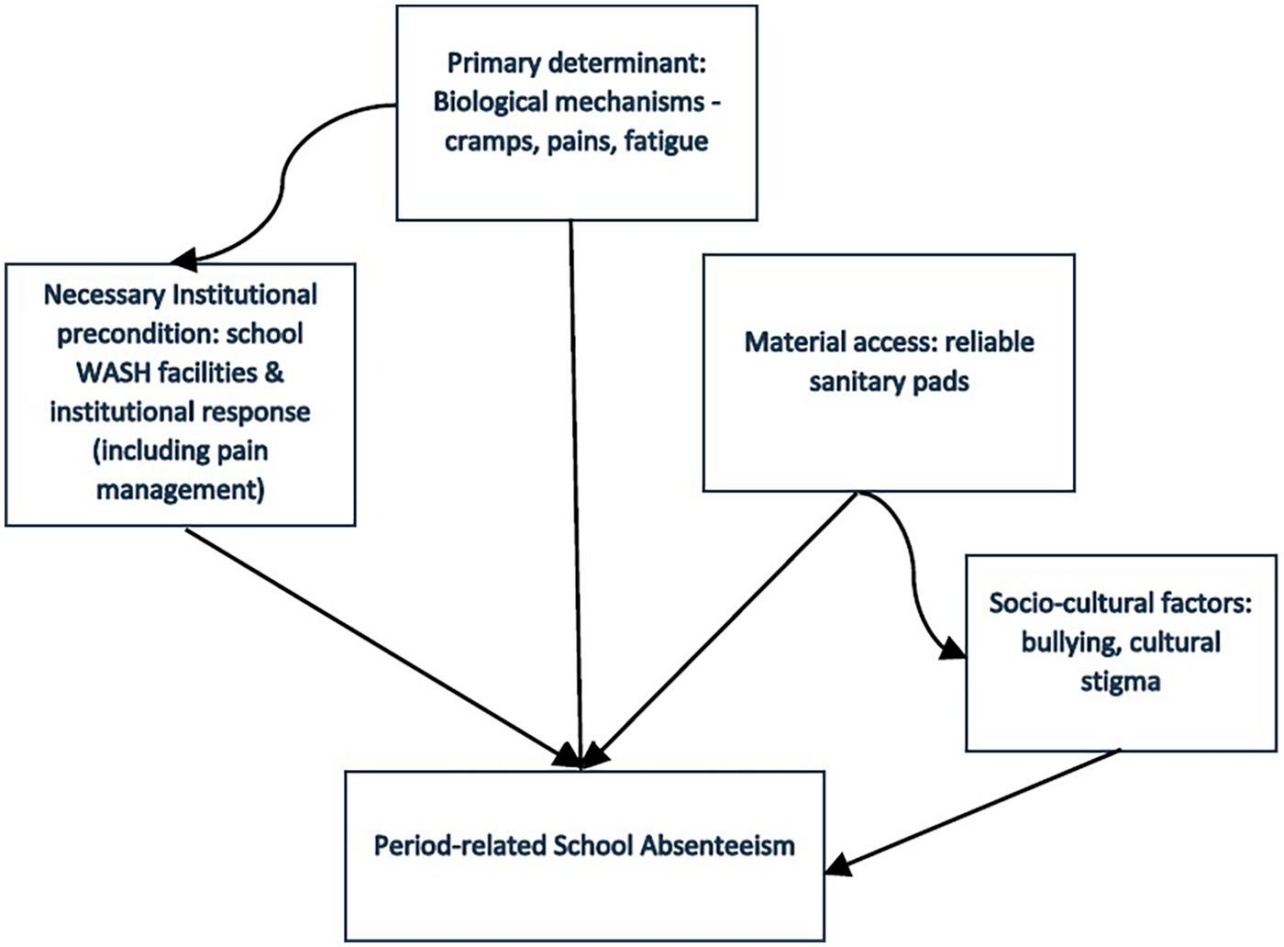
Determinants of period-related school absenteeism.

Taken together, the findings indicate that although the Government of Ghana's free sanitary pad initiative is widely regarded as timely and well-intentioned, its scope is inherently limited. It addresses only one pathway among several determinants of period-related absenteeism. This implies that the intervention cannot, by design, eliminate the problem it seeks to solve. The findings, therefore, highlight a mismatch between the complexity of period-related absenteeism and the narrow focus of current policy responses. We argue, nonetheless, that rather than viewing this as an implementation shortfall, it can be more accurately understood as a theory-of-change limitation. Our study shows that period-related absenteeism emerges from the interaction of material, infrastructural, biological, and socio-cultural constraints. Effective interventions are, therefore, likely to require bundled or integrated approaches that simultaneously address products, WASH facilities, menstrual discomfort, bullying, and stigma reduction. We illustrate this in the conceptual framework in Figure 5 below.

In sum, the proposed framework suggests that both theory and empirical research, which typically inform policy interventions, such as the case of Ghana's free sanitary pad initiative, must move beyond single-factor explanations of period-related absenteeism. Interventions that focus narrowly on one constraint, such as the provision of sanitary products, are unlikely to produce lasting change. Instead, future research should test integrated, multi-component strategies that address the biological, infrastructural, and social conditions that jointly shape girls’ ability to remain in school. Designing studies and policies around this broader ecology of constraints would better reflect the lived realities described by girls and increase the likelihood of meaningful and sustained reductions in absenteeism. And while this study is situated in Ghana, these mechanisms are unlikely to be Ghana-specific and instead reflect structural features common to many low- and middle-income school systems. Comparable challenges related to menstrual pain, inadequate sanitation facilities, and social stigma have been documented across Sub-Saharan Africa and South Asia. Ghana, therefore, serves less as an isolated case and more as an empirical illustration of a broader pattern. Viewed in this light, period-related absenteeism is best understood as a systemic or institutional failure rather than simply a shortage of products. This broader framing strengthens both the general relevance and the transferability of the findings beyond the immediate study setting.

Our study is, however, not without limitations. In particular, we acknowledge the potential challenges associated with our use of convenience sampling, as opposed to more probabilistic approaches such as simple random sampling, which would have afforded greater national representativeness and stronger claims regarding the generalizability of our findings. We recognize this as a potential weakness of the study design and therefore advise that the findings be interpreted in light of this limitation. Notwithstanding this, we believe the study offers important insights that can help inform and strengthen future interventions aimed at addressing period-related absenteeism in low-income and lower-middle-income countries.

## Data Availability

The dataset associated with this article can be accessed on Zenodo at: https://doi.org/10.5281/zenodo.20038022.

## Footnotes

^1^Given the nationwide standardization of Ghana’s public school system under the Ghana Education Service, selected districts are institutionally comparable to non-sampled districts.

^2^Field work took place between July -September 2025. The initial drafts of the data collection instruments—survey questionnaires and focus group discussion guides—were developed by the research team and shared with the GES for review and approval.

^3^We primarily used descriptive statistics in the quantitative analysis here because in a complementary paper, we estimate a series of multivariate logistic regression models to examine the independent association between menstrual discomfort, sanitation infrastructure, access to sanitary pads, and socio demographic factors with the likelihood of reporting period-related absenteeism more extensively to ascertain the explanatory power of access to sanitary pads on period-related absenteeism. Here, we estimate one supplementary ordinal logistic regression model as a robustness test for our qualitative findings.
